# Redefining Onyx HD 500 in the Flow Diversion Era

**DOI:** 10.1155/2012/435490

**Published:** 2011-10-27

**Authors:** Richard Tyler Dalyai, Ciro Randazzo, George Ghobrial, L. Fernando Gonzalez, Stavropoula I. Tjoumakaris, Aaron S. Dumont, Robert H. Rosenwasser, Pascal Jabbour

**Affiliations:** Department of Neurosurgery, Thomas Jefferson University Hospital, Philadelphia, PA 19107, USA

## Abstract

We report the largest US case series results using Onyx HD-500 (EV3), a new liquid embolic agent, in the successful treatment of 21 patients with wide-neck intracranial aneurysms (mean size 4.5 mm), which are at increased risk of incomplete occlusion or recanalization with standard endovascular intervention utilizing detachable platinum coils. All aneurysms were located in the anterior circulation, and three aneurysms presented as acute subarachnoid hemorrhages. Complete aneurysm occlusion was present in 19 of 21 patients (90%). On six-month followup, one patient with an initially small residual neck progressed to total occlusion. Aneurysm recanalization was not detected in any patients on mean follow up of 8.9 months in 11 patients. Four patients experienced transient neurologic deficits in the immediate postoperative period and one in a delayed fashion. Embolization with the liquid embolic agent Onyx appears to be a safe and effective endovascular modality of treatment for wide-neck aneurysms or recurrent aneurysms that had previously failed treatment with detachable coils.

## 1. Introduction

Treatment modalities achieving endosaccular occlusion of aneurysms continue to rapidly evolve. As technologies progress it is imperative to demonstrate improved efficiency, patient care, and clinical outcomes. Liquid embolic agents designed for aneurysm treatment potentially carry additional therapeutic benefits over coil embolization including improved volumetric aneurysm filling and endothelialization across the aneurysm neck which can lead to reduced recanalization rates with fewer procedures and lower expenses. The use of liquid embolic agents stands in contrast to the recent strategy of flow diversion for the treatment of intracranial aneurysms.

The Onyx liquid embolic system (EV3, Inc. Irvine, Calif, USA) is a combination agent of ethylene-vinyl alcohol copolymer, dimethylsulfoxide, and tantalum that has been primarily used in the endovascular treatment of arteriovenous malformations. Since 1999, Onyx has also been used in cerebral aneurysms and has been studied in several European and South American case series [1–3]. Since then, the technique for its use and types of polymers used have been refined with a newer high-viscosity agent, Onyx HD 500, designed specifically for the endovascular treatment of intracranial aneurysms. We report the largest published series of intracranial aneurysms treated in the United States with Onyx HD 500 for aneurysms with a wide neck (>4 mm or dome-to-neck ration <2).

## 2. Methods

This study was a US single center prospective registry study that included 21 consecutive patients with intracranial aneurysms treated by embolization with Onyx HD 500 between March 2008 and April 2011 in a single institution. Similar to patients enrolled in the CAMEO and other Onyx trials, patients were selected based on aneurysms characteristics that were difficult to treat by conventional methods of coil embolization with a neck wider than 4 mm or dome-to-neck ratios less than 2 [3]. Several aneurysms were also selected that had recurred following prior coil embolization. Data was collected on patients prior to the procedure, at the time of the procedure, at discharge, and again at 6–12-month followup. Clinical data that were evaluated included preoperative comorbidities and presenting symptoms, modified Rankin scale, cranial nerve deficits, any adverse effects, and changes in neurologic status as seen in Table 1.

All unruptured aneurysms were pretreated with clopidogrel 75 mg per day and aspirin 81 mg for at least 10 days before the procedure. Patients with subarachnoid hemorrhage (SAH) were loaded with 600 mg of clopidogrel during the procedure through the nasogastric tube. In only one patient was Enterprise stent (Codman Neurovascular, Raynham, MA, USA) assistance utilized at the time of onyx embolization; however, five patients were coiled previously, and one of them had a stent-assisted coiling that presented on follow up with a recurrence. In no patient was adjunctive coil embolization employed. The procedure time from microcatheter insertion to removal ranged from 25 to 120 minutes with a mean of 56 minutes. The average time under fluoroscopy was 75 minutes. The total volume of onyx used ranged from 0.2 mL to 8.4 mL with a mean of 1.2 mL.

**Figure 1:**
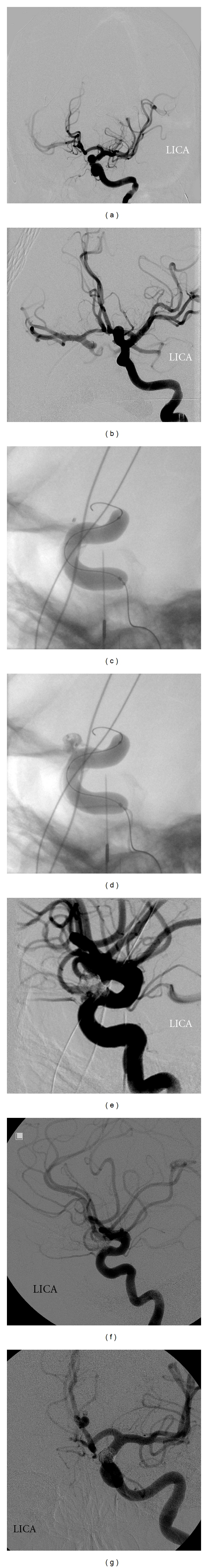
Angiograms showing the stages in the Onyx embolization of a 6.5 mm left ophthalmic artery aneurysm that was previously untreated. (a) Digital subtraction angiography (DSA) showing a 6.5 mm left ophthalmic artery aneurysm in the frontal and magnified frontal view (b). (c) Frontal projection of DSA showing “seal test” with Rebar 14 microcatheter in midportion of aneurysm with 4 × 30 Hyperglide balloon inflated. (d) Initial injection of Onyx HD 500. (e) Frontal and lateral. (f) DSA projection displaying immediate 95% occlusion with neck remnant. (g) Nine-month followup showing progression to complete occlusion in the frontal and lateral projections (h).

**Figure 2:**
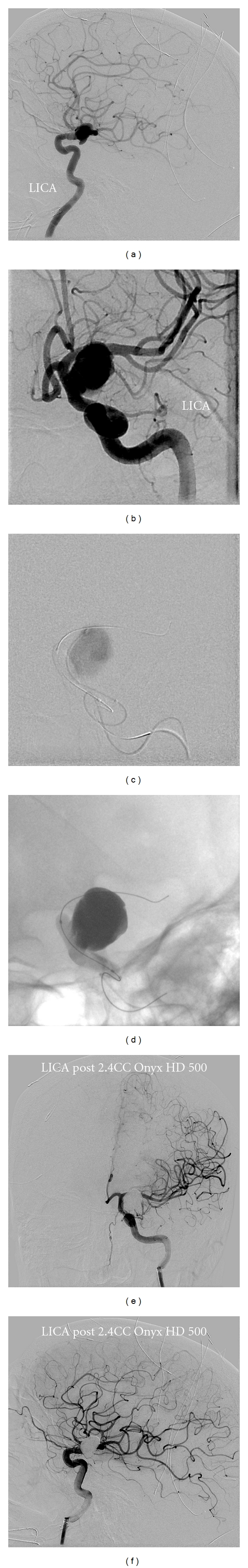
Angiograms displaying stages of Onyx embolization of an 85-year-old woman who presented with progressive vision loss and found to have 14 mm left ophthalmic artery aneurysm. (a) DSA showing a 14 mm L ophthalmic aneurysm in the Townes and Lateral (b) projections. (c) Magnified Townes projection showing 4 × 30 Hyperglide balloon inflated with contrast filling aneurysm. (d) Magnified frontal DSA projection showing Onyx filled aneurysm sac with complete filling. (e) DSA final runs showing completely occluded aneurysm in the frontal and lateral (f) projections.

All patients were treated under general anesthesia and full systemic anticoagulation with heparin. Since we perform all of our endovascular aneurysm treatments under general anesthesia, neurophysiological monitoring was used as a substitute for the neurological exam during these procedures. Electrophysiologic monitoring was performed for continuous BAERs, SSEPs, and EEG. Activated clotting times were maintained greater than 250 seconds throughout the entire case. Our technique used for Onyx injection is similar to those previously described as seen in Figures 1 and 2 [2–8]. After the Onyx embolic agent is dry heated and shaken, a 6F guide catheter is placed in the internal carotid artery and a DMSO-compatible Hyperglide balloon (EV3, Irvine, Calif, USA) positioned and inflated to allow its middle portion to cover the neck of the aneurysm. The microcatheters utilized in this study to access the aneurysm were the DMSO-compatible Rebar 14 (EV3) or Echelon 10, which are placed within the mid-portion of the aneurysm. Next, with the balloon inflated, contrast agent is slowly injected to ensure an effective seal test, demonstrating stasis of the contrast in the aneurysm and allowing estimation of the amount of balloon inflation needed for complete aneurysm occlusion while avoiding leakage of the embolic agent. The balloon is deflated at this point.

Next, saline is flushed through the microcatheter, and the dead space is filled with DMSO. Onyx is then carefully injected until the material approaches the end of the microcatheter at the recommended rate of 0.2 mL/2 min. With the DMSO then displaced, the balloon is inflated after 0.15 mL of onyx is injected to fill part of the microcatheter dead space. At this point, Onyx is injected to fill the aneurysm sac with the balloon inflated. In our institution, with the use of intraoperative neurophysiologic monitoring, the balloon is continuously inflated as long as the monitoring is stable. We do not interrupt our injection to deflate the balloon. Onyx is allowed to laminate around the balloon, creating an alpha sign, in the parent artery to cover the aneurysm neck, ensuring a complete and durable occlusion and reducing the risk of aneurysm recurrence. With confirmation of adequate aneurysm obliteration, there is a 10-minute hold period to allow complete solidification of the cast with the balloon deflated. The balloon is then inflated for counter resistance and the microcatheter removed by gentle but brisk traction.

## 3. Results

Patient's ages ranged from 35–85 years with a mean age of 57 as seen in Table 1. Three patients presented as acute Hunt and Hess Grade IV Subarachnoid Hemorrhage, 4 had a previous history of SAH, and the remaining 11 aneurysms were unruptured. Five patients presented with cranial nerve deficits. Of these 3 had cranial nerve III palsies from cavernous carotid aneurysms, and two had visual field deficits from ophthalmic artery aneurysms. Eleven patients presented with headaches, while 3 patients' aneurysms were completely incidental findings on MRI/A. Five patients had been previously treated with coil embolization but had recanalized, and 1 patient was previously coiled with stent assistance incompletely. All patients underwent cerebral digital subtraction angiography prior to treatment.

All of the aneurysms were located in the anterior circulation arising on a segment of the internal carotid artery. One aneurysm was located in the clinoid segment, 4 superior hypophyseal, 4 cavernous carotid, 5 posterior communicating, 6 ophthalmic, and 1 in the anterior choroidal segment. Ten aneurysms were located off the left ICA, while 9 were right sided. Fourteen aneurysms were small (<8 mm), six were large (8–24), and one was giant (≥24 mm). Eleven aneurysms had necks greater than 4 mm with a mean neck length of 4.5 mm. The average parent artery diameter was 4.3 mm. 

All 21 patients were successfully treated with Onyx HD 500, and 19 of 21 had complete occlusion as the immediate angiographic result with two patients displaying a small residual neck as seen in Table 2. One Grade IV Subarachnoid hemorrhage patient required decompressive hemicraniectomy postoperatively due to increased intracranial pressures, and another patient with a grade V SAH ultimately progressed to brain death. Three patients had minor unintentional embolic migration into the parent artery without flow limitation. On followup parent artery stenosis was not observed. Initially, 5 of the 21 patients experienced transient neurologic adverse events as described in Table 3. All patients undergoing their six-month followup had improved to their baseline neurologic condition. Ten of 21 patients (47%) had followup angiography within one year (one patient with follow-up Magnetic Resonance Angiography) and all of these patients achieved complete occlusion.

## 4. Discussion

Since the publication of the results of ISAT and ISUIA, the development of endovascular techniques utilizing platinum coils has been a dominant tool in the armamentarium of neurovascular interventionalists to achieve complete occlusion and prevent aneurysm rupture [9, 10]. However, aneurysms with wide necks (>4 mm), dome-to-neck ratios less than 1.5, or very large in dome height that have been treated with coils are associated with high rates of recanalization. This deficiency in the endovascular treatment of aneurysms has led to the development of newer techniques and materials to completely occlude these difficult-to-treat cerebral aneurysms.

Onyx liquid embolic is a biocompatible nonadhesive agent composed of ethylene-vinyl alcohol copolymer and dimethyl sulfoxide (DMSO) solvent added to tantalum powder to make the solution radioopaque. Being a liquid embolic agent allows Onyx to fill 100% of the aneurysm sac volume. Originally, different polymer concentrations Onyx 18 and 34 (6% and 8%) were used in AVM embolization. An Onyx 12% polymer concentration was then initially used for aneurysm embolization until Onyx HD (high density; 500 cP) was developed with a 20% copolymer concentration with an increased viscosity to lessen the likelihood of reflux into the parent artery. Onyx utilizes a balloon remodeling technique that may enable better control of the neck and parent vessel compared to traditional coiling [7]. There are also reports of Onyx inducing more neoendothelialization over the neck of the aneurysm relative to coils [6, 11].

There have been previous reports of the endovascular use of Onyx in intracranial aneurysms beginning with Mawad et al. describing embolization of 11 large and giant intracavernous aneurysms with an initial 82% complete occlusion and 91% at followup in 6 or 12 months [5]. They did not report any recanalization. The European CAMEO trial was the first multicenter trial with Onyx embolization that described 100 treated aneurysms with an occlusion rate at 1-year followup ranging from 93% in small aneurysms to 57% in giant aneurysms with an overall occlusion rate of 79% [3]. They retreated 10% of patients and reported an 8% permanent morbidity and 2% mortality rate. Following these trials with promising initial results, Onyx HD 500 was developed with a greater viscosity to reduce the risk of parent vessel occlusion and distal migration that were seen in these prior trials. Weber et al. was the first European trial to report their initial use Onyx HD 500 in 22 aneurysms with an immediate occlusion rate of 82% and 91% at approximately one year [8]. Temporary or minor complications were reported in 14% of patients, more permanently in 4.5% and 11% of aneurysms recanalized. They did not report any deaths. The largest South American Onyx HD 500 trial to date was published by Piske et al. which reported on 84 wide-neck aneurysms with a 66% total occlusion on short-term follow up, 85% at 6 months, and 90% at 18 months [6]. They reported progression from incomplete to complete occlusion in 68%, with recanalization in 5% in which 2 patients (3%) required retreatment. Their reported mortality was 2.9% with a permanent morbidity of 7%. Simon et al. was the first US group to discuss their preliminary experience with Onyx HD 500 in 12 intracranial aneurysms [7]. They described initial angiographic results in which all aneurysms were 90% or more occluded. All patients with followup six-month DSA displayed durable occlusion. They experienced one complication with ophthalmic occlusion and no mortalities.

Our results in 21 patients with wide-neck aneurysms treated with Onyx HD 500 are compared favorably with those of the CAMEO trial and other series and better than those reported for coil embolization as seen in Table 4. 19 patients achieved immediate complete occlusions, and one initially near-complete occlusion progressed to complete occlusion. Eight patients (38%) experienced adverse events following the procedure including worsening of preexisting cranial neuropathies in 2 patients, transient visual disturbance in 1 patient, TIAs in 1 patient, and femoral access complications in 1 patient; 2 patients with SAH had complications following the procedure that were due to their primary disease (1 grade IV patient required hemicraniectomy and 1 grade V patient died). Of the initial adverse events in unruptured aneurysms, none produced permanent morbidity with all neurological sequelae resolving completely at discharge or on followup.

These results are encouraging and must be weighed against recent aneurysm treatment strategies utilizing endoluminal treatments and flow diversion. These techniques aim to induce aneurysmal thrombosis by diverting the flow away from the aneurysm; however, this obliteration effect takes place in a delayed fashion and requires weeks to months for full effect. In the setting of acute SAH, flow diversion is relatively contraindicated when considering that the risk of rerupture is close to 20% in 2 weeks and 50% by 6 months. Also, flow diversion stents can not be used at arterial bifurcations. Further, with early reports on the Pipeline stent describing some delayed aneurysm ruptures and intra parenchymal hemorrhages flow diversion stent's efficacy and adverse effect profile cannot be assumed until larger series are reported [12].

**Figure 3:**
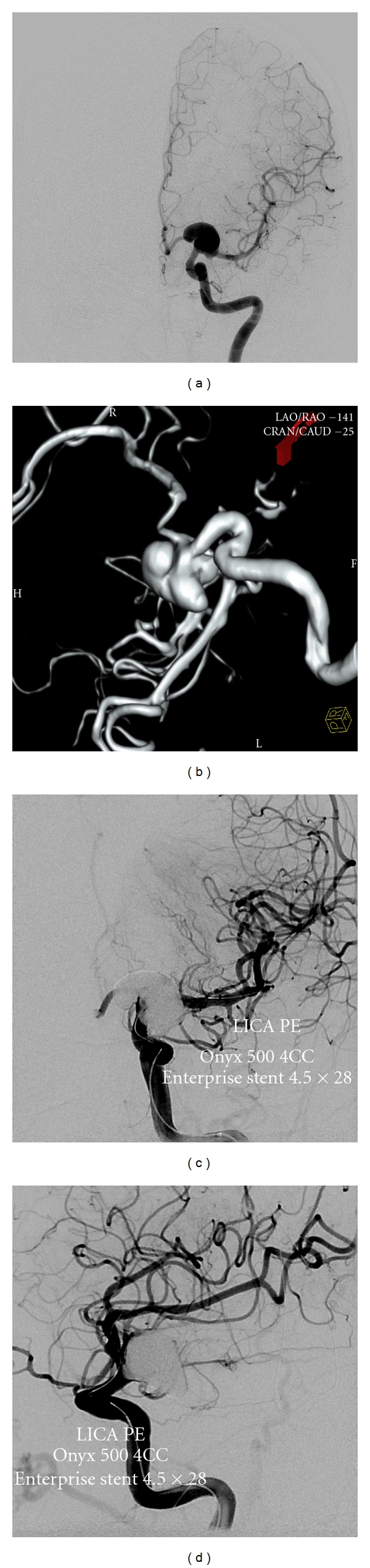
Angiograms demonstrating stages of Onyx embolization of a 53-year-old man presenting with severe headaches and found to have 19 × 13 mm left anterior choroidal aneurysm with 5 mm neck as seen in the A/P (a) and 3D reconstruction (b). (c) DSA showing magnified frontal and lateral. (d) Projection after Onyx embolization and placement of Enterprise stent.

At this point in time, Onyx HD 500 has still some specific indications, such as multilobulated aneurysms close to a bifurcation (like our case in Figure 3), wide-neck aneurysms below 10 mm (considering the FDA has only approved the pipeline embolization device for aneurysms >10 mm), the setting of subarachnoid hemorrhage, in previously stented aneurysms where pipeline cannot provide the diversion desired, and in small recurrences after coil embolization where Onyx HD 500 can effectively seal the neck of the aneurysm.

In our series, we treated three SAH patients (2 grade IV and one grade V) and immediately achieved complete aneurysm occlusion in all patients. Two patients were eventually discharged to inpatient rehabilitation facilities with permanent disabilities, and the other ultimately proceeded to brain death. Similar to flow diversion techniques however, Onyx embolization requires antiplatelet agents preoperatively and postoperatively, making its use in patients with an acute aneurysm rupture less than ideal. However, the morbidity and mortality of an otherwise unprotected and recently ruptured aneurysm and the lack of other options at the time (based upon aneurysm characteristics) made the use of Onyx HD 500 a necessity. 

Taken together, our overall results using Onyx HD 500 were encouraging, but will necessarily need to be confirmed with larger series and in comparison to newer self-expanding stent-assisted coiling and flow diversion techniques.

## 5. Conclusion

Onyx HD 500 appears to be a safe and effective endovascular modality of treatment for wide-neck aneurysms that are not amenable for, or had previously failed treatment with detachable coils. With an adequate patient selection, this method may be a better alternative than coiling in regards to packing density, intraoperative rupture, and recurrence rate. In the flow diversion era, Onyx HD 500's use needs to be redefined; however it remains a valuable tool in the endovascular armamentarium.

## Figures and Tables

**Table 1:** 

Case no.	Procedure date	Age	Sex	Presenting symptoms	Hx of SAH	Pre-Op mRS	Aneurysm location	Aneurysm side	Type of aneurysm	Aneurysm dome height (mm)	Aneurysm dome width (mm)	Aneurysm neck length (mm)	Parent artery diameter (mm)
1	03/18/08	45	F	Incidental	None	0	Ophthalmic artery	Left	Sidewall	4.4	4.1	5.5	4.5
2	03/18/08	54	F	Incidental	Previous SAH	0	Posterior communicating	Right	Sidewall	3.6	2.6	2.4	4.0
3	05/13/08	83	F	Aneurysm rupture/SAH	SAH	5	Posterior communicating	Right	Sidewall	10.0	12.1	7.5	4.4
4	10/10/08	54	F	Aneurysm rupture/SAH	SAH	5	Superior hypophyseal	Right	Sidewall	3.4	4.3	4.4	5.7
5	11/17/09	35	F	Headache	None	0	Ophthalmic	Left	Sidewall	6.0	5.0	4.0	4.0
6	12/09/09	44	F	Incidental	Previous SAH	0	Posterior communicating	Right	Sidewall	3.0	3.0	3.0	4.8
7	12/21/09	61	F	Occulomotor nerve palsy	None	0	Cavernous carotid	Right	Sidewall	20.0	15.0	8.0	3.8
8	12/23/09	35	F	Headache	None	0	Superior hypophyseal	Right	Sidewall	4.0	3.0	2.5	4.5
9	12/24/09	59	M	Occulomotor nerve palsy	None	1	Cavernous carotid	Left	Sidewall	27.0	18.0	7.0	5.0
10	01/26/10	50	F	Incidental	Previous SAH	0	Posterior communicating	Left	Sidewall	3.0	2.2	2.0	4.0
11	01/29/10	57	F	Visual field deficit	None	0	Ophthalmic	Left	Sidewall	6.0	6.5	4.5	4.0
12	04/28/10	54	F	Incidental	Previous SAH	0	Posterior communicating Artery	Right	Sidewall	2.8	3.0	2.0	3.9
13	09/24/10	85	F	Visual field deficit	None	1	Ophthalmic	Left	Sidewall	12.0	14.0	4.5	4.3
14	10/07/10	68	F	Headache	None	0	Clinoid segment	Right	Sidewall	8.0	12.0	6.6	4.0
15	10/20/10	60	F	Occulomotor nerve palsy	None	0	Cavernous Carotid	Left	Sidewall	8.0	9.0	3.0	2.0
16	11/01/10	68	F	Aneurysm rupture/SAH	None	5	Cavernous carotid	Right	Dissecting	5.0	4.0	5.0	4.0
17	11/19/10	52	F	Headache	None	0	Ophthalmic	Left	Sidewall	5.3	8.0	9.2	5.0
18	02/28/11	66	F	Headache	None	0	Superior hypophyseal	Left	Sidewall	5.6	4.5	3.0	4.5
19	04/04/11	53	F	Headache	AVM	1	Opthalmic	Left	Sidewall	5.1	5.6	3.3	4.5
20	04/18/11	69	F	Headache	None	0	Superior hypophyseal	Right	Sidewall	3.9	3.3	3.9	5.0
21	04/21/11	53	M	Incidental	None	0	Anterior choriodal	Left	Sidewall	5.7	4.5	3.5	4.0

**Table 2:** 

Case no.	Plavix 650 mg given	Balloon used	Microcatheter	Access catheter	Total volume (mL) Onyx injected (incl dead space)	Immediate angiographic Raymond scale	Immediate occlusion	Adverse events	mRS at discharge
1	Yes	4 × 30 Hyperglide	Rebar 14	8F Guiding catheter	0.30	Complete occlusion	100%	No adverse events	0
2	Yes	4 × 30 Hyperglide	Rebar 14	8F Guiding catheter	0.30	Complete occlusion	100%	No adverse events	0
3	No	10 × 30 Hyperglide	Rebar 14	8F Guiding catheter	0.50	Complete occlusion	100%	Required decompressive hemicraniectomy	5
4	Yes	4 × 30 Hyperglide	Rebar 14	8F Guiding catheter	0.20	Complete occlusion	100%	Death	6
5	Yes	4 × 30 Hyperglide	Rebar 14	8F Guiding catheter	0.70	Complete occlusion	100%	No adverse events	0
6	Yes	4 × 30 Hyperglide	Rebar 14	8F Guiding catheter	0.60	Small residual neck	95–99%	Femoral access complication	0
7	Yes	4 × 30 Hyperglide	Rebar 14	8F Guiding catheter	4.80	Complete occlusion	100%	Worsening of oculomotor deficit	0
8	Yes	4 × 30 Hyperglide	Rebar 14	8F Guiding catheter	0.80	Complete occlusion	100%	TIAs	0
9	Yes	4 × 30 Hyperglide	Rebar 14	8F Guiding catheter	8.40	Complete occlusion	100%	Worsening of oculomotor deficit	1
10	Yes	4 × 30 Hyperglide	Rebar 14	8F Guiding catheter	0.50	Complete occlusion	100%	No adverse events	0
11	Yes	4 × 30 Hyperglide	Rebar 14	8F Guiding catheter	0.85	Small residual neck	95–99%	TIAs	1
12	Yes	4 × 30 Hyperglide	Rebar 14	8F Guiding catheter	0.43	Complete occlusion	100%	Transient visual disturbance	1
13	Yes	4 × 30 Hyperglide	Rebar 14	8F Guiding catheter	2.70	Complete occlusion	100%	No adverse events	1
14	Yes	5 × 30 Hyperglide	Echelon 10	8F Guiding catheter	1.40	Complete occlusion	100%	Asymptomatic Cervical ICA dissection	0
15	Yes	3 × 10 Hyperglide	Echelon 14	8F Guiding catheter	1.20	Complete occlusion	100%	No adverse events	0
16	No	5 × 30 Hyperglide	Echelon 10	6F Guiding catheter	0.55	Complete occlusion	100%	No adverse events	5
17	Yes	5 × 20 Hyperglide	Echelon 10	6F Guiding catheter	0.35	Complete occlusion	100%	No adverse events	0
18	Yes	5 × 20 Hyperglide	Echelon 10	6F Guiding catheter	0.30	Complete occlusion	100%	No adverse events	0
19	Yes	5 × 30 Hyperglide	Rebar 10	6F Guiding catheter	0.40	Complete occlusion	100%	No adverse events	0
20	Yes	4 × 30 Hyperglide	Echelon 10	6F Guiding catheter	0.30	Complete occlusion	100%	No adverse events	0
21	Yes	5 × 30 Hyperglide	Echelon 14	8F Guiding catheter	0.40	Complete occlusion	100%	No adverse events	0

**Table 3:** 

Case no.	Timing of followup	Degree of change of followup angiogram	Followup raymond scale	Followup angio % occlusion	Followup mRS
1	9 months	No change	Complete occlusion	100%	0
2	7 months	No change	Complete occlusion	100%	0
6	12 months	No change	Complete occlusion	100%	0
7	9 months	No change	Complete occlusion	100%	0
8	11 months	No change	Complete occlusion	100%	0
9	6 months	No change	Complete occlusion	100%	1
10	8 months	No change	Complete occlusion	100%	0
11	9 months	Increased occlusion	Complete occlusion	100%	0
12	12 months	No change	Complete occlusion	100%	0
14	6 months (MRA)	No change	Complete occlusion	100%	0

**Table 4:** 

Authors	Date published	Journal	# Aneurysms treated	Primary outcome	% Mortality	% Permanent disability attributed to procedure
Mawad et al. [5]	Mar, 2002	J Neurosurg	11	GOS	9%	9%
Molyneux et al. [3]	Jan, 2004	AJNR	97	Percent occlusion	2%	8%
Lubicz et al. [2]	Apr, 2005	AJNR	41	mGOS	3%	7%
Weber et al. [8]	Sept, 2005	AJNR	22	Percent occlusion	0%	0%
Cekirge et al. [1]	Jan, 2006	Neuroradiology	100	mRS	3%	8%
Piske et al. [6]	May, 2009	Neurosurgery	84	Percent occlusion	3%	7%
Simon et al. [7]	Sept, 2010	Neurosurgery	12	Durable occlusion	0%	8%
